# Supplementary figures and images for: TTLL12 has a potential oncogenic activity, suppression of ligation of nitrotyrosine to the C-terminus of detyrosinated α-tubulin, that can be overcome by molecules identified by screening a compound library
Source: PLoS One. 2024 Feb 23;19(2):e0296960. doi: 10.1371/journal.pone.0296960 (PMC10889654; doi:10.1371/journal.pone.0296960)

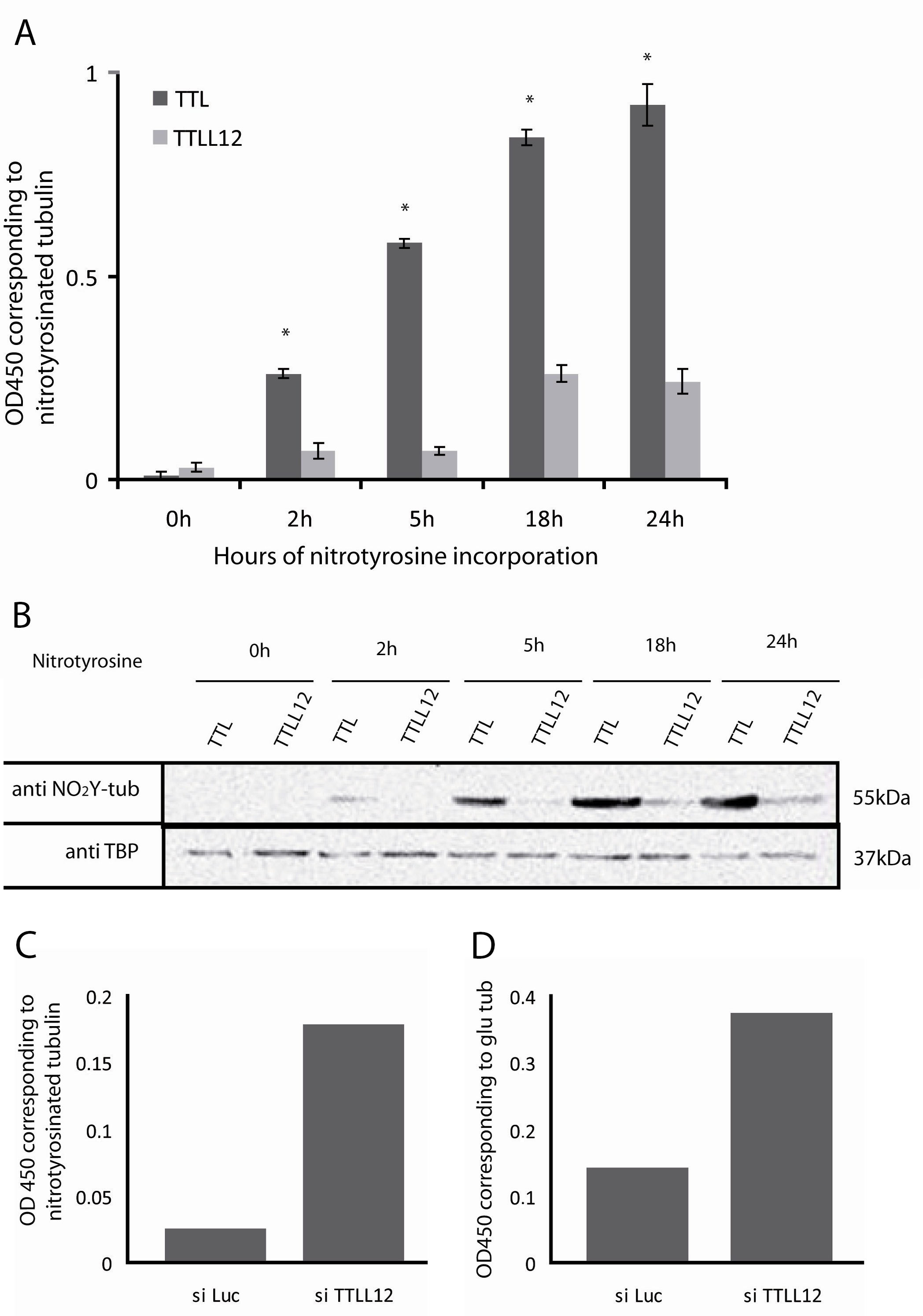

Supplement: S1 Fig — (A) The optimized cell-based ELISA-HRP detects the difference in α-tubulin nitrotyrosination between TTL (positive control) and TTLL12 clones (screening line). HEp-2 cells were treated with 400 μM nitrotyrosine for 0–24 h and C-ELISA-HRP was performed as described in the Materials and Methods. The error bars represent the ± SEM of three wells in one experiment (* indicates Z’ factors > 0.5). (B) Western blots confirm the differences in α-tubulin nitrotyrosination and increase with time for both the clones. The TTL and TTLL12 clones were treated with medium supplemented with 400 μM nitrotyrosine for 0–24 h, total lysates were prepared at the indicated times and analyzed by immunoblotting with anti-nitrotyrosine and anti-TBP. (C) C-ELISA-HRP detects increased levels of nitrotyrosinated α-tubulin induced by TTLL12 knockdown. HEp-2 cells were transfected with 10 nM siTTLL12 or si-luciferase and seeded in a 96-well plate. C-ELISA-HRP using nitrotyrosinated tubulin as the readout was performed as described in the Materials and Methods. The Z’ factor was > 0.5. (D) C-ELISA-HRP detects increased levels of glu-tubulin induced by TTLL12 knockdown. HEp-2 cells were transfected with siTTLL12 or si-luciferase and seeded in 96-well plates. C-ELISA-HRP using glu-tubulin as the readout was performed as described in the Materials and Methods. Z’ factor = 0.3. (TIF) [file pone.0296960.s001.tif]

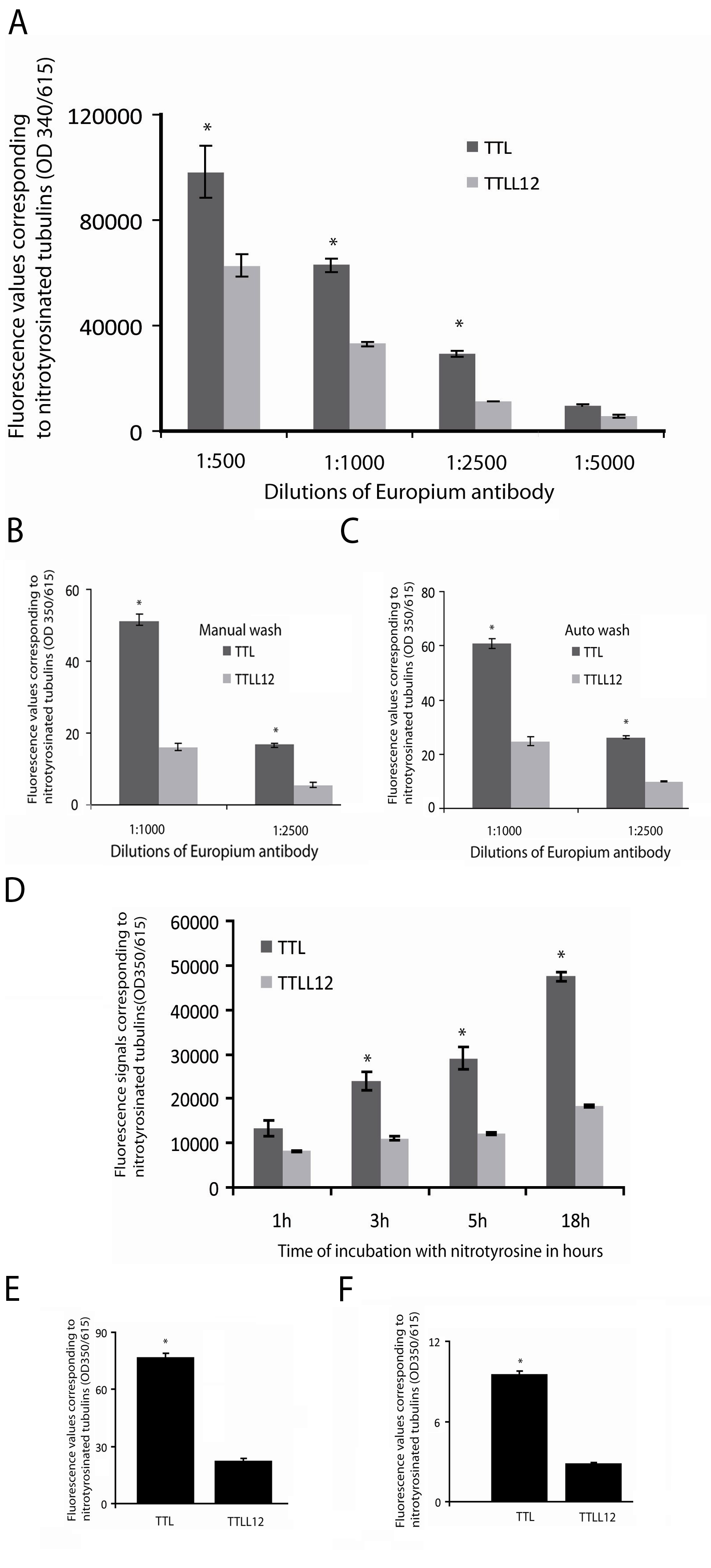

Supplement: S2 Fig — (A) Europium conjugated secondary antibodies can be used for the cell-based assay. C-ELISA was performed with Europium conjugated rabbit IgG at different dilutions. The error bars represent the ± SEM of three wells in one experiment (*Z’ factor > 0.5). (B, C) The washing steps were done either manually (B) or with an automated wash system (C). The error bars represent the ± SEM of three wells in one experiment (* Z’ factor > 0.5). (D) C-ELISA-Eu detects the increase in α-tubulin nitrotyrosination with time. The error bars represent the ± SEM of three wells in one experiment (*Z’ factor > 0.5). (E, F) Washing steps with PBS can be replaced with the DelphiaTM buffers used for the C_ELISA-Eu. (E) Washing steps before the addition of rabbit IgG were done with PBS, and DELPHIATM buffer was used thereafter. (F) All the washing steps were performed with DELPHIATM buffer. The error bars represent the ± SEM of three wells in one experiment. The y-axis represents the time-resolved fluorescence values at emission/excitation wave lengths of 340/615 nm. The symbol * indicates Z’ factors > 0.5. (TIF) [file pone.0296960.s002.tif]

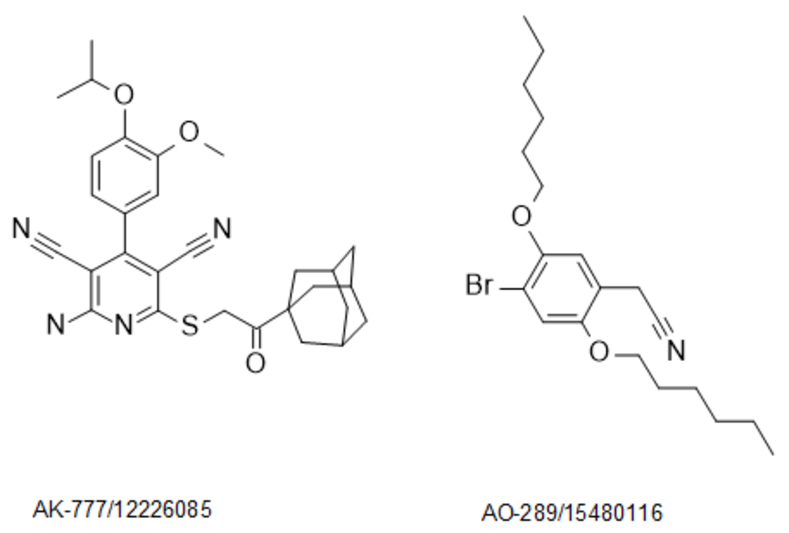

Supplement: S3 Fig — (TIF) [file pone.0296960.s003.tif]
